# A Novel Mouse Model for Non-Invasive Single Marker Tracking of Mammary Stem Cells *In Vivo* Reveals Stem Cell Dynamics throughout Pregnancy

**DOI:** 10.1371/journal.pone.0008035

**Published:** 2009-11-25

**Authors:** Benjamin J. Tiede, Leah A. Owens, Feng Li, Christina DeCoste, Yibin Kang

**Affiliations:** Department of Molecular Biology, Princeton University, Princeton, New Jersey, United States of America; Roswell Park Cancer Institute, United States of America

## Abstract

Mammary stem cells (MaSCs) play essential roles for the development of the mammary gland and its remodeling during pregnancy. However, the precise localization of MaSCs in the mammary gland and their regulation during pregnancy is unknown. Here we report a transgenic mouse model for luciferase-based single marker detection of MaSCs *in vivo* that we used to address these issues. Single transgene expressing mammary epithelial cells were shown to reconstitute mammary glands *in vivo* while immunohistochemical staining identified MaSCs in basal and luminal locations, with preponderance towards the basal position. By quantifying luciferase expression using bioluminescent imaging, we were able to track MaSCs non-invasively in individual mice over time. Using this model to monitor MaSC dynamics throughout pregnancy, we found that MaSCs expand in both total number and percentage during pregnancy and then drop down to or below baseline levels after weaning. However, in a second round of pregnancy, this expansion was not as extensive. These findings validate a powerful system for the analysis of MaSC dynamics *in vivo*, which will facilitate future characterization of MaSCs during mammary gland development and breast cancer.

## Introduction

The mammary gland is a highly dynamic organ, undergoing well choreographed growth and involution during development, estrous cycles, and pregnancy [1], [2]. In pubertal mice, a structure named the terminal end bud invades into the empty mammary stroma, also known as the mammary fat pad. Once fully developed, the mammary gland forms a ductal tree structure composed of branches with hollow lumens capped at the ends by secretory acinar structures. During pregnancy, the mammary epithelium expands extensively, filling in much of the mammary fat pad, and then undergoes involution after weaning to return to a state similar to the pre-pregnant gland. The mammary gland is composed of two primary cell types: luminal epithelial and myoepithelial cells. Luminal epithelial cells line the inside of the ducts and are believed to be derived from the inner body cells of the terminal end bud. A subset of these luminal cells is responsible for secreting milk during pregnancy. Contractile myoepithelial cells on the other hand are believed to be derived from the outer cap cells of the terminal end bud and exist on the external layer of the ducts. Despite many years of research, the identity of the mouse mammary epithelial stem cell (MaSC), capable of differentiating into these cell types and responsible for gland formation and remodeling, was not elucidated until recently [3], [4].

Using fluorescence activated cell sorting (FACS) and mammary fat pad transplantation experiments, MaSC-enriched populations were identified by the expression of CD24 along with high expression of either CD29 or CD49f [3], [4]. Subsequent research has just begun to elucidate what governs MaSCs growth and differentiation as well as how MaSCs function in the mammary gland [5], [6], [7], [8]. Studying MaSC regulation *in vivo* is critical for understanding stem cell-niche interactions in the mammary gland and investigating the potential link between MaSC activity and breast cancer susceptibility. Unfortunately, the high degree of precision in detecting protein level variations using FACS cannot be achieved in immunostaining and thus has made it difficult to localize MaSCs by immunostaining of surface markers in combination. Furthermore, even after definitive identification of MaSCs, the only currently available method to monitor their dynamics was to use flow cytometry after dissecting mammary glands, making it impossible to monitor MaSCs in individual mice over time.

In order to overcome these difficulties, we have characterized a novel mouse model for *in vivo* MaSC tracking based on our discovery that MaSC-enriched cells from a luciferase/GFP-transgenic mouse strain [9] are the only mammary epithelial cell type with appreciable transgene expression. Single cells, sorted based solely on the expression of the transgene were able to repopulate mammary glands *in vivo*. By monitoring luciferase expression in recipient mice, we were able to quantitatively track the regulation of MaSCs in individual mice non-invasively using bioluminescence imaging (BLI). Additionally, the restricted expression of luciferase allowed us to precisely identify the location of MaSC-enriched epithelial populations in the mammary gland using immunohistochemistry.

## Results

### Restricted Luciferase Expression in Functional MaSCs

**Figure 1:**
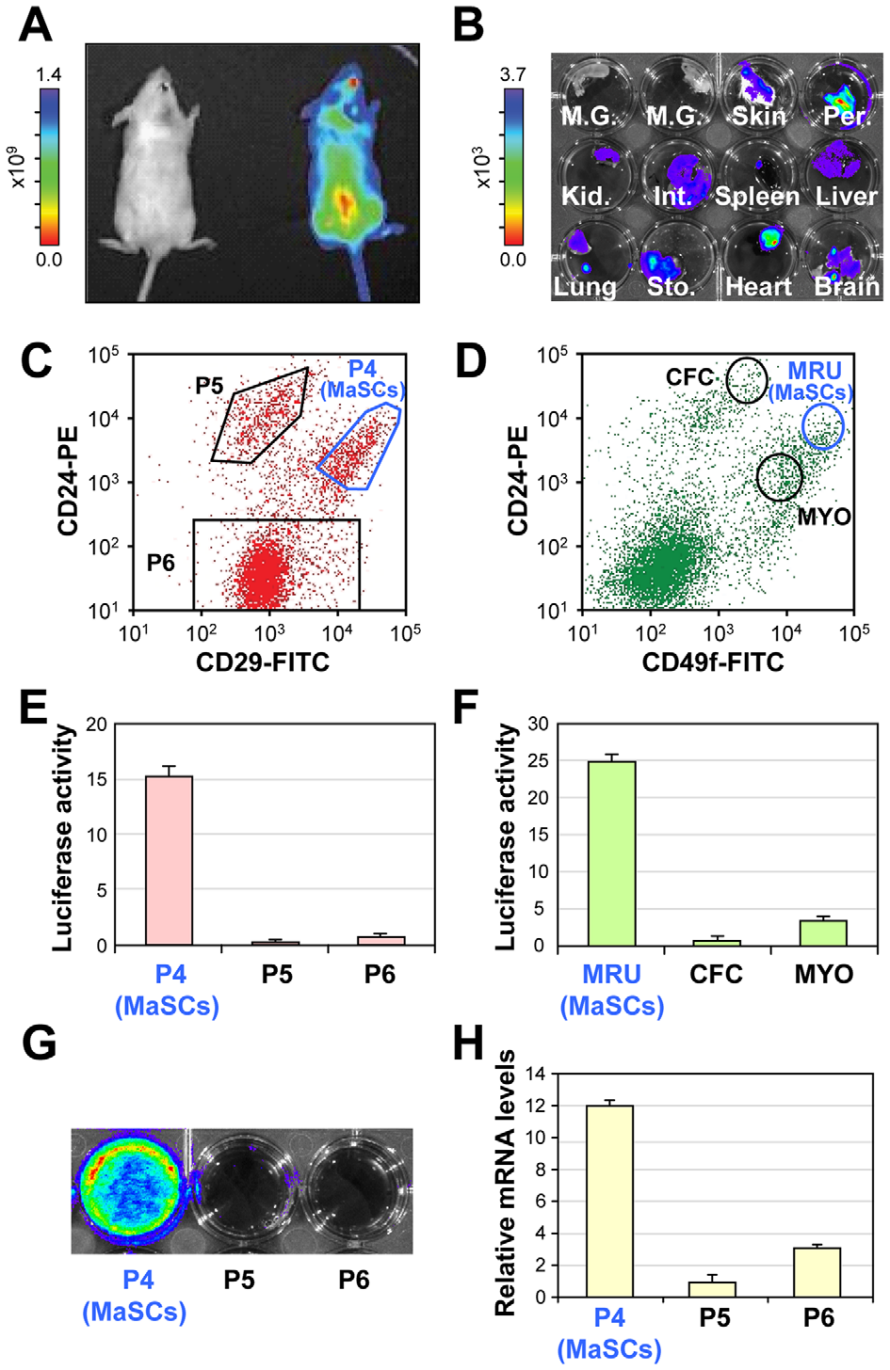
Luciferase activity from the mammary glands of a luciferase-transgenic mouse is restricted to mammary stem cells. (**A**) Representative bioluminescence (BLI) images of FVB-N/J control and luciferase transgenic mice. (**B**) BLI images of individual organs from luciferase transgenic animals. Luciferase activity is largely absent from the mammary glands of luciferase transgenic mice. Abbreviations: M.G., mammary gland; Per., peritoneum; Kid., kidney; Int., intestine; Sto., stomach. (**C,D**) Mammary glands were dissociated and stained with CD24 and either CD29 (**C**) or CD49f (**D**) (MRU: Mammary Repopulating Unit, equivalent to MaSC; CFC: Colony Forming Cells; MYO: Myoepithelial cells) before subjecting to flow cytometry analysis and sorting. (**E, F**) Sorted MaSCs showed elevated luciferase expression compared to other mammary epithelial populations (data normalized to the luciferase activity of unsorted mammary epithelial populations +/− SEM). (**G**) Representative BLI images of sorted populations based on CD24 and CD29 staining. (**H**) qRT-PCR analysis of *luciferase* mRNA levels (+/− SEM) normalized to the levels of *α-tubulin*.

When comparing the relative luciferase expression in different organs from transgenic mice designed to express luciferase and GFP under the control of the *CMV* enhancer/*β-actin* promoter [9], the mammary gland showed significantly lower expression of luciferase than other organs from the same mouse (Fig. 1A, B). After dissociating the gland into a single cell suspension and sorting using either of the two published MaSC surface marker profiles (CD24^+^CD29^hi^ or CD24^+^Cd49f^hi^) [3], [4], ∼15–25 fold higher expression of luciferase was observed in the MaSCs compared to the other mammary epithelial populations (Fig. 1C–G) which mirrored the levels of *luciferase* mRNA (Fig. 1H). This restricted pattern of luciferase expression is stable across many generations of mice.

**Figure 2:**
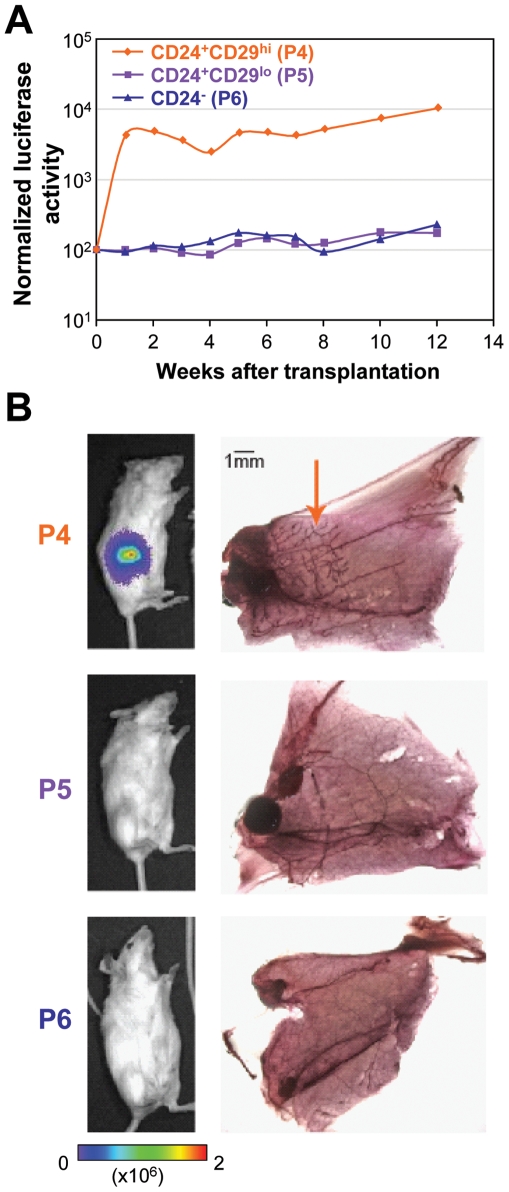
Luciferase activity correlates with *in vivo* stem cell activity. (**A**) 1000 mammary epithelial cells of the indicated population were transplanted into mammary fat pads cleared of their endogenous epithelium and subsequent BLI intensity was quantified in individual mice over 12 weeks, normalized to the time of injection. (**B**) Representative BLI images (left panel) and alum carmine staining of mammary glands (right panel) 12 weeks after transplantation of various mammary epithelial cell populations into cleared fat pads. Luciferase activity was detected solely in mice receiving CD24^+^CD29^hi^ MaSCs (P4) and not in mice receiving CD24^+^CD29^lo^ (P5) and CD24^−^ P6 cells (middle and lower panel). Mice with strong luciferase activity showed mammary gland reconstitution, based on alum carmine staining (arrow).

In order to test whether the luciferase expressing MaSC enriched populations possessed functional stem cell activity *in vivo*, we performed cleared fat pad mammary gland reconstitution assays. Using the Lin^−^CD24^+^CD29^hi^ surface marker profile, the MaSC-enriched population, along with putative mammary committed progenitor cells (Lin^−^CD24^+^CD29^lo^) and other mammary epithelial cells (Lin^−^CD24^−^) were sorted from luciferase transgenic mice and transplanted into non-transgenic mammary fat pads cleared of their endogenous epithelium. Luciferase expression, which expanded quickly within the first two weeks after transplantation before reaching a relative steady state, was detected solely in the mice receiving CD24^+^CD29^hi^ cells (Fig. 2A). Representative images of recipient mice are shown in Fig. 2B, along with alum carmine staining of the transplanted mammary fat pads. Of critical importance is that whenever luciferase expression was detected in recipient mice, mammary gland reconstitution was also observed (Fig. 2B - orange arrow), indicative of functional *in vivo* stem cell activity.

**Figure 3:**
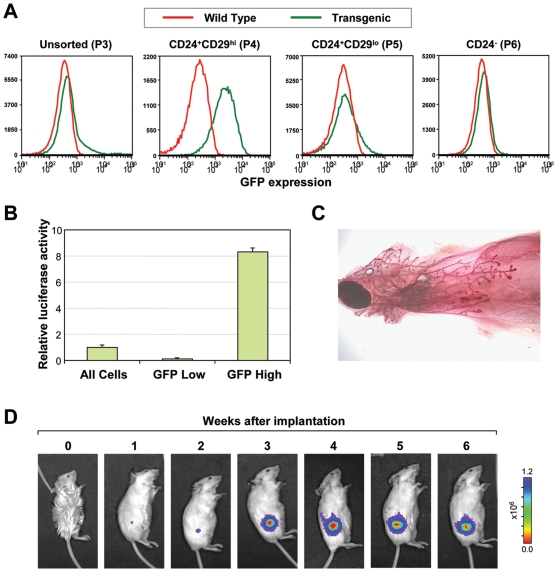
GFP positive cells can reconstitute a mammary gland. (**A**) Flow cytometry analysis of GFP expression in the indicated mammary epithelial populations based on CD24 and CD29 staining in wild type (red) and luciferase transgenic (green) mice. A noticeable shift in the CD24^+^CD29^hi^ (P4) population in GFP expression is observed (second panel), which accounts for the small GFP^hi^ shoulder in the unsorted cells (first panel). (**B**) When GFP^hi^ and GFP^lo^ cells are collected and tested for luciferase expression, luciferase activity is largely restricted to the GFP^hi^ population. (**C**) Confirmation of single cell reconstitution of the mammary gland by alum carmine staining of the mammary gland in 4 of 52 recipient mice. (**D**) BLI images of a representative mouse after receiving transplantation of a single GFP^hi^.

Because not every cell in the FACS-sorted populations (Fig. 1C, D) is a MaSC, the possibility arose that the luciferase expressing cells within these MaSC-enriched populations may be distinct from the functional MaSC population. To address this, we sought to test the reconstitution ability of single luciferase expressing cells. However, since FACS is unable to sort live cells based on the expression of luciferase, we investigated whether the luciferase expressing CD24^+^CD29^hi^ cells also expressed any detectable GFP. Even though the bulk of the mammary gland, like other tissues from the adult transgenic mouse [9], did not show much GFP signal, we saw a small but distinct GFP signal in the CD24^+^CD29^hi^ population (Fig. 3A). By sorting Lin^−^ cells solely based on the GFP expression, luciferase expression was highly restricted to the GFP^hi^ subpopulation (Fig. 3B). To definitively show that the luciferase/GFP expressing cells were functional stem cells, single visualized GFP^hi^ cells were transplanted into cleared mammary fat pads. In 4 of 52 transplants, mammary gland reconstitution was observed (Fig. 3C, D), which is a similar rate to published cell surface markers [3]. Additionally, by transplanting 500 GFP^hi^ and 4000 or 10,000 GFP^lo^ cells, we observed that MaSC activity was highly enriched in the GFP^hi^ population (Fig. S1). Based on these results, we are confident that luciferase expression could indeed serve as a single, robust marker for MaSC activity both *in vivo* and *in situ* to address biological questions concerning the regulation and localization of MaSCs.

### Immunohistochemical Localization of MaSCs

**Figure 4:**
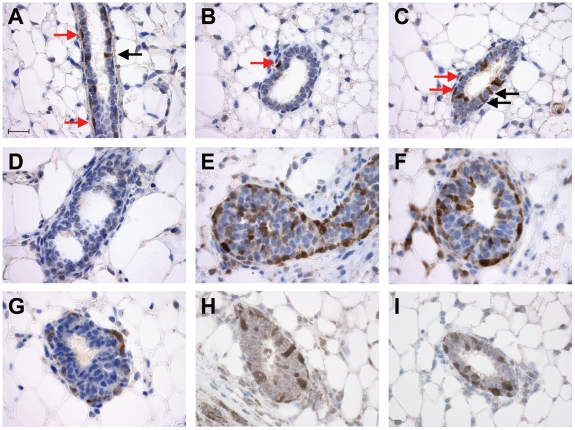
Localization of luciferase expressing MaSCs in the mammary gland. (**A–C**) Immunohistochemical staining of 4µm paraffin sections of mammary glands from a 12-week-old luciferase transgenic mouse. Luciferase expressing MaSCs are observed in two locations: a basal position, indicated by red arrows, and a luminal position, indicated by black arrows. (**D**) Control staining of luciferase in non-transgenic mouse. (**E,F**) Staining of 3-week-old luciferase transgenic mouse mammary glands revealed an increased number of luciferase expressing MaSCs in terminal end buds. (**G–I**) Reconstituted glands from 1000 sorted CD24^+^CD29^hi^ P4 cells (**G**) singe GFP^+^ (**H**) or 500 GFP^+^ (**I**) sorted cells all showed that the luciferase expressing transplanted cells were found to differentiate into non-luciferase expressing cells as they reconstitute the gland. Scale bar in (**A**) represents 20µm. All panels in this figure use the same scale.

By performing immunohistochemical staining against the luciferase protein, we were able to clearly localize the MaSC-enriched cells within the mammary gland (Fig. 4). In 12-week-old nulliparous mice, luciferase expressing cells were seen in two distinct locations: a basal position between the myoepithelial and luminal epithelial compartments (Fig. 4A–C, red arrows), and a luminal position (Fig. 4A–C, black arrows). Among the 9.0% of luciferase expressing cells, 6.3% of the cells were located in the basal position while 2.7% percent were in the luminal position. While the presence of luciferase expressing cells in the luminal position was initially unexpected, it is interesting to note that CD29 deletion from the basal compartment of the mammary gland was recently found to ablate a significant amount of MaSC function, but did not prevent the formation of secretory acinar structures during pregnancy [11], suggesting the possibility of a distinct stem/progenitor population outside the basal layer. This is also consistent with the finding that in the MaSC-enriched fraction isolated by CD24 and CD49f, there was a subpopulation of cells which expressed the luminal marker Keratin 18 [4]. When mammary glands from younger transgenic mice are stained (∼3 weeks old), an overall higher number of luciferase-positive MaSC-enriched cells is observed, both in the cap and ductal regions of the terminal end buds (Fig. 4E, F)

Importantly, when luciferase expressing MaSCs isolated by CD24^+^CD29^hi^ or GFP expression are transplanted they differentiate into mostly non-luciferase expressing mammary epithelial cells (Fig. 2) as they reconstitute the mammary gland. The reconstituted mammary glands show similar numbers of luciferase positive and negative cells to glands of transgenic mice (Fig. 4G–I). Thus, by tracking luciferase dynamics *in vivo* rather than *in situ*, we are confident we can monitor MaSC activity in real time during physiologically relevant processes.

### Dynamics of MaSCs throughout Pregnancy

**Figure 5:**
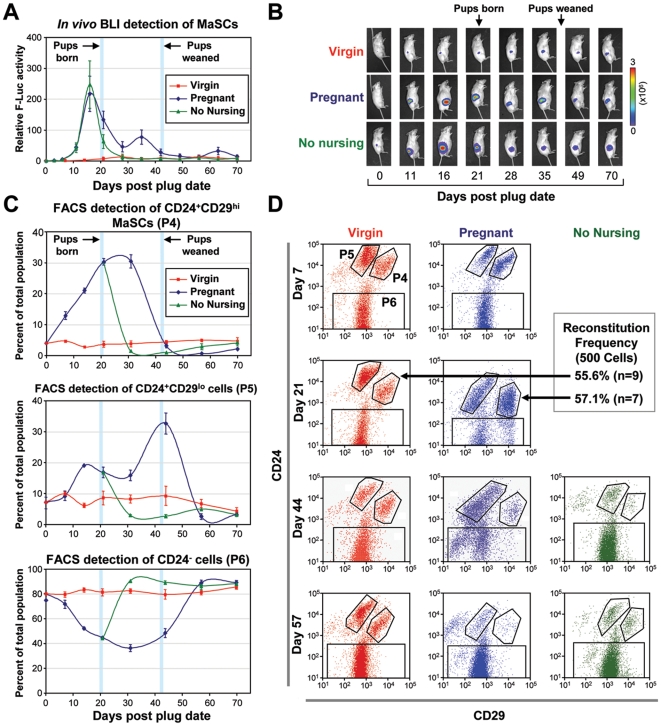
Mammary epithelial cell dynamics throughout pregnancy. (**A**) Average (+/− SEM) of luciferase activity in five pregnant (blue) and seven virgin (red) mice after cleared fat pad injections (FPI) where endogenous epithelium was removed prior to transplantation. A separate group of seven mice which did not nurse their pups are represented in green. Day 0 represents the day that mice in the pregnancy group were plugged, and the days that pups were born and weaned are highlighted by the blue bars. (**B**) Representative BLI images of mice from the pregnant, virgin, and no nursing groups. (**C**) The kinetics of MaSCs (P4), progenitors (P5) and other epithelial cells (P6) in pregnant (blue) and virgin (red) mice was detected using flow cytometry. Mice that did not nurse their pups are again shown in green. Each data point represents the average percentage of each population of the total mammary epithelium from four to six mice per experimental group. (**D**) Representative flow cytometry analysis results of various mammary epithelial populations in mice from the pregnant, virgin and no nursing groups at different time points of the experiment. The reconstitution ability of P4 cells from virgin and pregnant mice around the peak in number of P4 cells during pregnancy is roughly equal as highlighted by the black arrow.

The major function of the mammary gland is to supply milk during nursing. In order to perform this function, the gland undergoes a massive expansion to generate secretory structures to produce milk. The regulation of this expansion was believed to be due to stem cell activity, but this has not been shown directly. Previous studies have attempted to quantify the overall change in the number of MaSCs after pregnancy [12], [13]. However, these studies only looked at one time point well after pups were weaned and showed differing results based on the age of mice. To further investigate the activity of MaSCs during the entire course of pregnancy, two to three weeks after luciferase expressing MaSCs were transplanted into fat pads of 3 week old mice cleared of their endogenous epithelium (at which point their activity reaches a relative steady state – see Fig. 2A), we induced pregnancy and monitored luciferase activity to detect MaSC dynamics. On average, MaSC activity rose roughly 200 fold during pregnancy and began to drop back down immediately after the pups were born, eventually returning close to baseline levels around the time that pups are weaned (Fig. 5 A,B). This effect is clearly dependent on the mother nursing their young pups, as MaSC numbers drop precipitously if the pups are separated right after birth (Fig. 5, “No Nursing” group).

We also monitored MaSC regulation using the traditional flow cytometry method (which quantifies the changes in percentage of cells within the mammary gland rather than total number) by sacrificing mice at each individual time point throughout the pregnancy cycle. Comparing the two methods allowed us to asses the possibility that the expansion and regression in the number of MaSCs observed *in vivo* by BLI may simply correlated with the changes in the gland as a whole, since the mammary gland expands extensively in response to pregnancy and involutes after weaning. We observed a similar expansion and regression of MaSCs based on percentage (Fig. 5C, D), suggesting that MaSCs expansion proceeded in a faster rate than the gland as a whole. Using flow cytometry also permitted us to quantify the changes in the putative mammary progenitors (Lin^−^CD24^+^CD29^lo^) and other mammary epithelial cells (Lin^−^CD24^−^). Intriguingly, the percentage of CD24^+^CD29^lo^ cells both rose and fell with a delayed kinetics following the expansion and regression of CD24^+^CD29^hi^ cells, indicating that these cells may be direct progenies of CD24^+^CD29^hi^ cells as has been suggested elsewhere [14]. The number of CD24^+^CD29^lo^ cells also dropped precipitously when mothers did not nurse their pups (Fig. 5C). As CD24^+^CD29^lo^ cells have been shown to contain lactogenic precursors, it is not surprising that their expansion is observed after pregnancy, during nursing.

**Figure 6:**
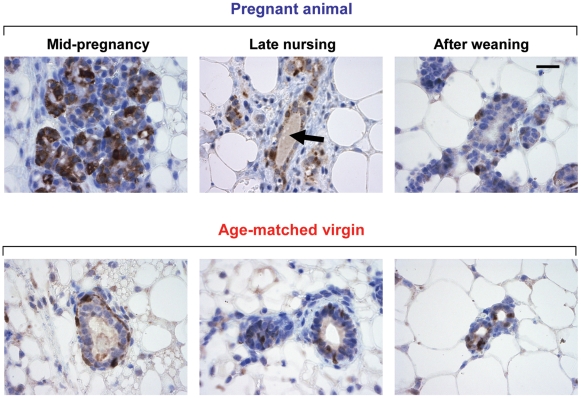
Luciferase immunohistochemistry of parous glands. Mammary glands were taken from pregnant mice midway through their first pregnancy, at the end of the nursing of their pups, and roughly one month after pups were weaned as well as from age matched virgin controls. Strong luciferase staining can be seen both in the alveolar bunches of the parous glands, as well as in the ductal regions and in the milk (black arrow). After pups are weaned, the amount of luciferase expressing MaSCs returns back to similar levels as aged matched virgin controls. Scale bar represents 20µm.

To better observe the morphological changes associated with the massive expansion of MaSC activity, we performed immunostaining of luciferase transgenic mammary glands throughout pregnancy (Fig. 6). While it is clear that the number of luciferase expressing cells rises in both the alveolar buds and the mature ducts during pregnancy, precise quantification of the changes in cell numbers was difficult because of the weakening of gap junctions that typically occurs during pregnancy, which results in comparatively diffuse staining [1]. After pups are separated from their mothers, the number of luciferase positive cells in the mammary gland returns back to similar levels of age matched virgin controls.

While we clearly observed an expansion of luciferase positive cells, and in the number of CD24^+^CD29^hi^ cells, this did not prove that the expansion was the result of an actual increase of MaSCs, since it could also be due to the expansion of another luciferase expressing cell population within the CD24^+^CD29^hi^ population. To address this, we first performed luciferase assays on sorted mammary epithelial cells from the luciferase transgenic mice just after giving birth to pups (near the peak in both number and percentage of MaSCs) and observed a restricted pattern of luciferase expression in CD24^+^CD29^hi^ MaSCs similar to virgin controls (Fig. S2). Additionally, we performed mammary fat pad reconstitution assays with CD24^+^CD29^hi^ cells from pregnant and virgin mice at a limited dilution (500 cells) where we expected not every mouse would show reconstitution. If the parity related rise in CD24^+^CD29^hi^ luciferase expressing cells were the result of an expansion of a non-MaSC population, we would expect to see less reconstitution when transplanting the same number of cells from pregnant and virgin mice. However, we observed similar levels of reconstitution (57.1% for pregnant, n = 7 and 55.6% for virgin, n = 9) suggesting that the rise in luciferase expressing CD24^+^CD29^hi^cells was indeed a rise in MaSCs.

**Figure 7:**
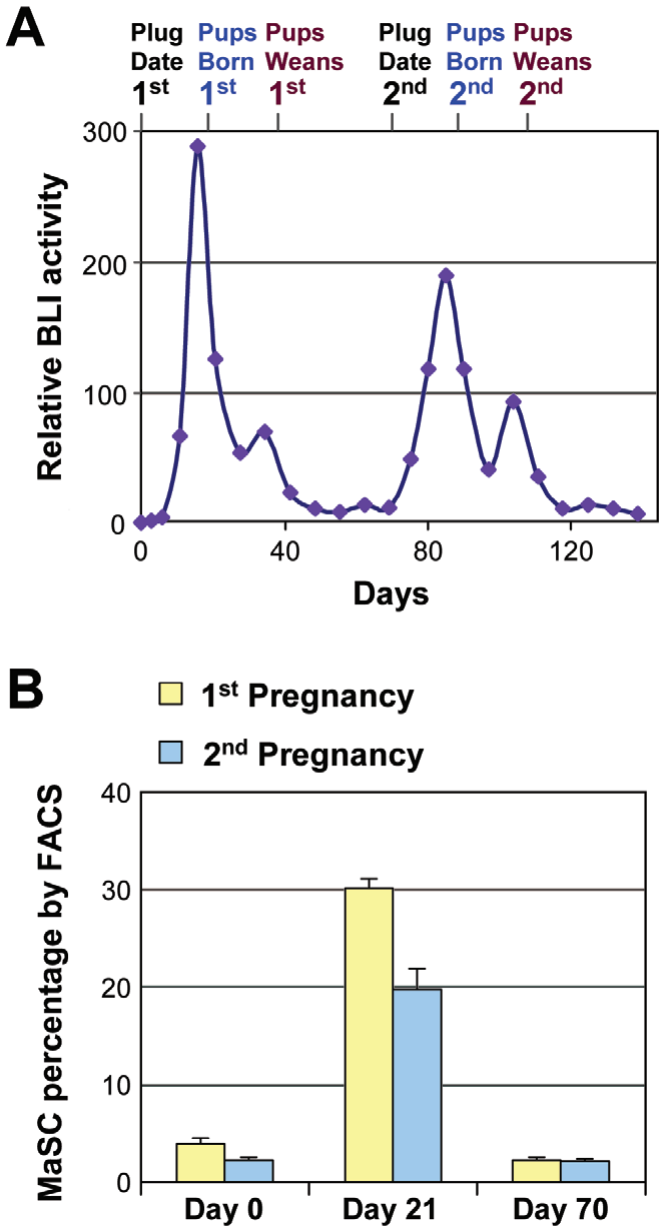
MaSC kinetics during two consecutive pregnancies. (**A**) MaSC activity detected by BLI in a single representative mouse through two pregnancies. (**B**) Flow cytometry data from three time points for mice in their first or second pregnancy. Each data point represents the average percentage (+/− SEM) of the mammary gland of P4 cells from four mice.

In order to better understand the regulations of MaSCs during pregnancy, we also assessed the regulation of MaSCs throughout a second round of pregnancy using both flow cytometry and the BLI methods (Fig. 7). Because tissue collection for flow cytometry requires sacrificing of donor mice, averages must be compared from different mice across pregnancies. Our non-invasive model has the advantage of being able to monitor the kinetics in individual mice throughout successive pregnancies. Overall there was a moderate reduction in the peak amount of MaSCs during the second pregnancy when normalized to plug date, which was evident in both our *in vivo* model (Fig. 7A) and using flow cytometry (Fig. 7B). The number of MaSCs returned back to similar levels after weaning of pups.

## Discussion

The isolation of mouse MaSCs capable of forming a functional mammary gland from a single cell has opened up many new avenues of research to study the function and regulation of these cells. These avenues will diverge down many paths, from studying the role of MaSCs in mammary gland development, to understanding the regulation of self renewal and differentiation of MaSCs, to investigating a potential role of MaSC in breast tumorigenesis. Recent studies have begun to unveil the complex nature of MaSC regulation. For example, CD29 (β1 integrin) and the Wnt receptor Lrp5 are found to be important for MaSC function [11], [15], while the differentiation of MaSCs has been shown to be influenced by the transcription factor GATA-3 [14] and Notch signaling [7], [8]. Furthermore, the growth of MaSCs is likely to be directly regulated by epidermal growth factor via binding to EGFR/ErbB1 but only indirectly by estrogen or progesterone since they do not express their respective receptors [5], [6]. Despite these recent advances, better methods to sensitively and non-invasively monitor MaSC activity *in vivo* are urgently needed to facilitate future study of MaSC function and regulation. The model we have presented in this study will be a valuable research platform in the characterization of MaSCs since it is the only tool available for quantitative real-time tracking of MaSC-enriched cells in living animals. Few if any other adult stem cell models allow for both single marker histological analysis of adult stem cell localization *and* the ability to monitor adult stem cell activity in individual mice over time. Our model represents a significantly simplified surrogate to the more cumbersome FACS methods which can not be used to assess MaSC activity longitudinally in living animals and requires a large number of animals to obtain data at different time points of the experiment. Using this model in combination with immunohistochemistry, we have identified the location of luciferase expressing MaSCs in both a luminal and basal compartment. Future characterization will help to elucidate whether these two epithelial populations are functionally distinct from one another. Furthermore, this model can be coupled with genetic manipulations of MaSCs *in vivo* or *ex vivo* [16] prior to transplantation or with manipulation of the host microenvironment to study how MaSCs are regulated by intrinsic signaling pathways or extrinsic cues from their surrounding niche.

We have also used this model to monitor MaSC dynamics throughout pregnancy, where we have shown that MaSCs rise in both total number and percentage during pregnancy and then decline to or below baseline levels after weaning. This effect is dampened when mothers do not nurse their young. Additionally, the magnitude of MaSC expansion is decreased during a second pregnancy. These results are particularly interesting when considered in the context of breast cancer, as it is known that in both mice and humans, full-term and multiple pregnancy results in a short term increase, but ultimately a long term decrease in breast cancer susceptibility [17]. It has been proposed that this is due to the transient expansion of MaSCs during pregnancy and their depletion afterwards, as MaSCs or progenitor cells may serve as the particularly susceptible cellular targets for transformation. Curiously, this protective effect is weakened when mothers do not nurse their young. Future studies should directly test the susceptibility of MaSCs to transformation to better understand how changes in their cell number throughout pregnancy may alter the likelihood of developing breast cancer.

In summary, our model serves as a unique platform in which MaSC activity can be non-invasively, sensitively and quantitatively monitored by bioluminescence imaging. This system will not only allow for the monitoring of MaSC activity in normal physiologically relevant processes but will also permit more direct evaluation of the susceptibility of MaSCs to oncogenic transformation and the regulation of their growth throughout tumorigenesis. If MaSCs play a critical role in the initiation or progression of breast cancer, this model will serve as an ideal system to develop and test future therapeutic applications that target MaSCs, including potentially novel prophylactic breast cancer treatments for high risk groups.

## Materials and Methods

### Animal Studies

All experiments involving mice were performed in accordance with approved protocols by the Institutional Animal Care and Use Committee of Princeton University. FVB/N-J mice were purchased from The Jackson Laboratory, FVB/N-Luciferase transgenic mouse strain L2G85 [9] were a gift of Dr. Chirstopher Contag and Dr. Yu-an Cao.

### Mammary Epithelial Cell Preparation and FACS

Single cell suspensions were prepared from the mammary glands of luciferase transgenic animals after mechanical and enzymatic dissociation based on published protocols [3], [4]. Briefly, mammary glands were excised, minced using scalpels, and digested for 1hr in 300U/ml type 1A collagenase (Sigma) and 100U/ml hyaluronidase (Sigma). Cells were then treated with 0.25% trypsin/EDTA, dispase (Invitrogen)/DNase (Sigma), and ammonium chloride in succession. Between each treatment, cells were rinsed in MEGM (1∶1 DMEM∶F12 Ham supplemented with 5µg/ml insulin, 500ng/ml hydrocortisone, 10ng/ml EGF, 20ng/ml cholera toxin, 5% bovine calf serum, and 1× penicillin/streptomycin). Afterwards, cells were resuspended in FACS buffer (PBS supplemented with 5% newborn calf serum plus 1× penicillin/streptomycin) and filtered twice through 40µm nylon cell strainers. The following antibodies were used to label cells: biotin conjugated anti-TER119 (red blood cells, BD cat#553672), biotin conjugated anti-CD31 (endothelial cells, BD cat#558737), biotin conjugated anti-CD45 (hematopoietic cells, BD cat#553078), CD29-FITC (Serotec, cat#MCA2298F), CD49f-FITC (BD cat#555736) and CD24-PE (BD cat#553262). Allophycocyanin (APC) conjugated Streptavidin (BD cat#554067) was used for secondary staining of lineage markers. For all staining, 50µl of antibody diluted 1∶75 in FACS buffer was used per 1×10^6^ cells, including single color controls and combination staining. Control samples for triple color FACS included no-staining, propidium iodide (PI, cell viability dye) only, CD24-PE only, CD29-FITC only, Biotin-lineage only as well as corresponding PE, FITC and APC single color fluorochrome conjugated antibody isotype controls. Both primary and secondary staining was conducted for 30 minutes at room temperature in FACS buffer. Between staining, cells were washed with 5ml FACS buffer. Cell sorting for transplantation experiments was done using a FACSVantage SE w/DiVa (BD Biosciences), whereas flow cytometric analysis of mammary epithelial cell populations during pregnancy was performed on a four color, dual-laser FACSort instrument (BD Biosciences). All cells were sorted into 1∶1 Fetal Bovine Serum∶MEGM.

### In Vitro Luciferase Assays

Sorted cells were centrifuged for 5 minutes at 1000g and then resuspended in 1× lysis buffer and incubated for 1hr at room temperature. Substrate was added and luciferase was detected using a GLOMAX microplate reader and a firefly luciferase assay kit (Promega). All samples were assayed in triplicate.

### qRT-PCR Analysis

RNA was isolated from sorted mammary epithelial populations using the Trizol (Invitrogen) and contaminating DNA was removed using the Ambion DNA-free kit. cDNA synthesis was performed using the Superscript III First-Strand Synthesis System for RT-PCR (Invitrogen). qRT-PCR was run on an ABI 7900 96 well machine using the PowerSYBR Green PCR Master Mix from Applied Biosystems. Samples were run in duplicate using the following primers: *Tubulin* Forward: CCTTCATTGGAAACAGCACA, *Tubulin* Reverse: CCTCCTCTCCGAAATCCTCT; *Actin* Forward: GTATCCATGAAATAAGTGGTTACAGG, *Actin* Reverse: GCAGTACATAATTTACACAGAAGCAAT, *Luciferase* Forward: ATCACAGAATCGTCGTATGC, *Luciferase* Reverse: GAAATCCCTGGTAATCCGTT

### Mammary Fat Pad Transplantation and Bioluminescent Imaging

After FACS, cells were centrifuged for 5 minutes at 1000g and then resuspended in 50% Matrigel/50% PBS. Mice were anesthetized by intraperitoneal injection of ketamine (100mg/Kg) and xylazine (10mg/Kg). Unless otherwise noted, all injections involved placing 1000 sorted mammary epithelial cells into cleared inguinal (#4) mammary fat pads according to standard injection procedures [10]. For single cell injections, cells live cells were detected using trypan blue staining and visualized in 10µl Terasaki wells prior to injection. Weekly bioluminescent imaging was done by injecting anesthetized mice with 100µl of luciferin solution (15mg/ml) through the orbital plexus. Luciferase activity was measured *in vivo* using the Xenogen IVIS 200 imaging system.

### Alum Carmine Staining of Mammary Glands

Excised mammary glands were fixed for 1 hour in 3∶1 glacial acetic acid: 100% ethanol, washed in 70% ethanol for 15 minutes and stained in alum carmine overnight at room temperature. Glands were then washed for 15 minutes in 70%, 95% and 100% ethanol and transferred for long term storage into HistocClear II clearing agent (National Diagnostics).

### Immunostaining

Histology services were performed by the Histopathology Core Facility at Brigham and Women's Hospital. Formalin fixed paraffin embedded mammary glands were sectioned (4µm), incubated at 58°C for 1 hour and then deparaffinized and rehydrated by washing in twice in xylene, 100% ethanol, 95% ethanol and dH_2_O. Antigen retrieval was performed using a pressure cooker (Biocare Medical) at peak temperature of 125°C with EDTA, pH 8. Slides were washed with Tris buffer with Tween-20 before applying peroxidase block (Dako) for 5 minutes, rinsing with 1× Tris, and bathing in serum-free protein block (Dako) for 20 minutes. Primary staining against luciferase was done with 1∶5000 to 1∶10000 diluted Novus goat polyclonal anti-firefly luciferase antibody (NB100-677) in Dako Antibody diluent for 1 hour followed by secondary staining with 1∶500 diluted rabbit anti-goat (Dako) for 30 min. Slides were then treated with Dako envision anti-rabbit for 30 min, and then treated with DAB before counterstaining with hematoxylin.

## Supporting Information

Figure S1 Enriched MaSC activity in GFPhi cells. (A) 500 GFP^hi^ cells were injected into 16 recipient mice while 4000 or 10,000 GFP^lo^ cells were injected into 8 mice per group. BLI activity was subsequently measured over six weeks and normalized to the signal at the time of injection. (B) Reconstitution ability was highly enriched in the GFP^hi^ fraction, with successful reconstitution (green dots) in 9 out of 16 recipients, compared to 3/8 mice receiving 10,000 GFP^lo^ cells and 1/8 mice receiving 4000 GFP^lo^ cells. Overall, the 14 cases of reconstitution that were confirmed by alum carmine staining also represented the top14 final BLI readings.
(9.30 MB TIF)

Figure S2 *In vitro* luciferase assay for sorted mammary epithelial cells from virgin and parous mice. Mammary epithelial cell populations were collected based on CD24 and CD29 staining and lysed for in vitro quantification of luciferase activity. Data shown is normalized to P4 cells (+/− SEM). The same pattern of luciferase activity is seen in virgin and pregnant mice, with the higher expression of luciferase in unsorted populations as the result of the higher percentage of luciferase expressing MaSCs in the parous mice.
(3.76 MB TIF)
