# Supplementary figures and images for: A Novel Mouse Model for Non-Invasive Single Marker Tracking of Mammary Stem Cells In Vivo Reveals Stem Cell Dynamics throughout Pregnancy
Source: PLoS One. 2009 Nov 25;4(11):e8035. doi: 10.1371/journal.pone.0008035 (PMC2777504; doi:10.1371/journal.pone.0008035)

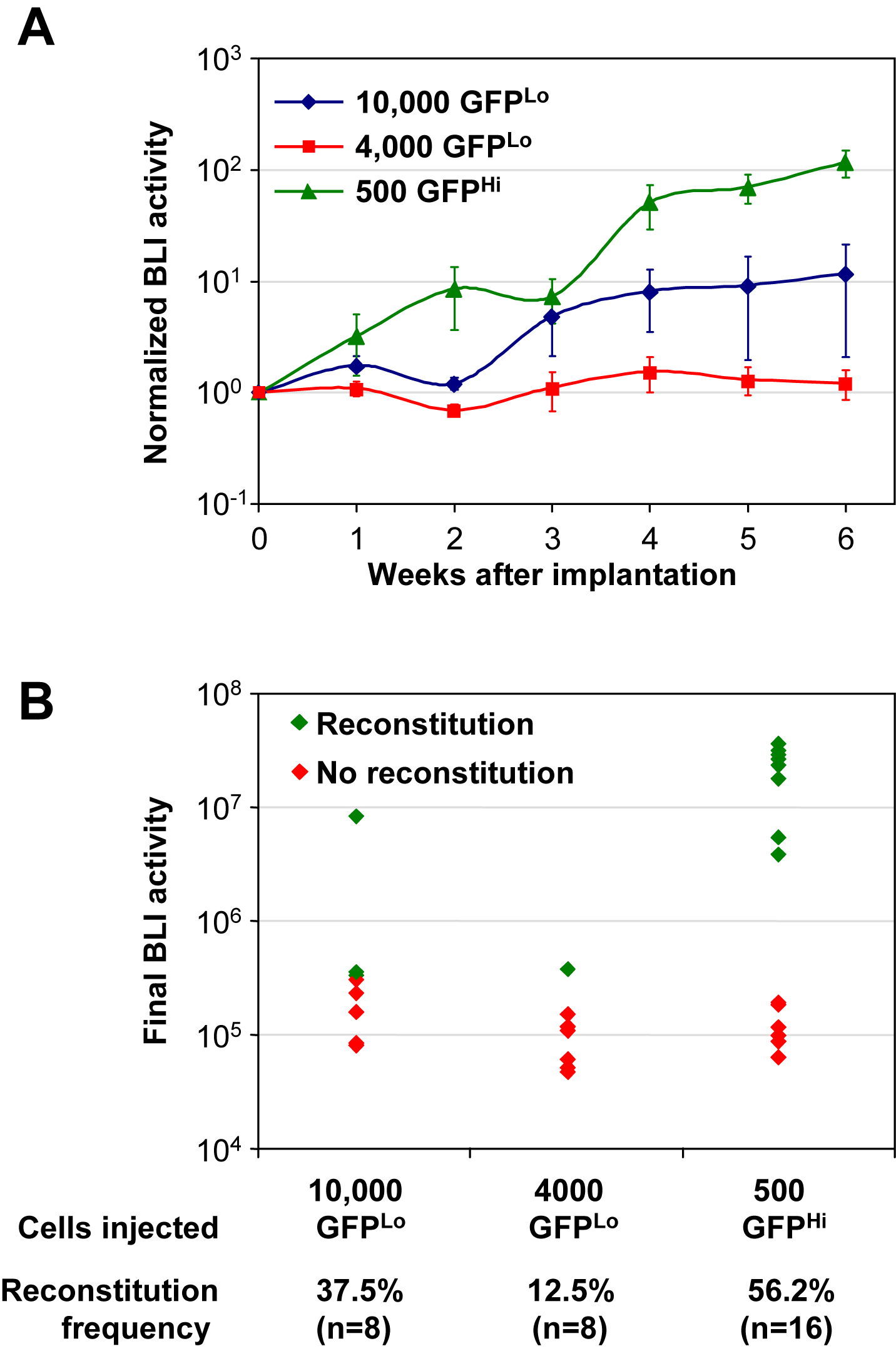

Supplement: Figure S1 — Enriched MaSC activity in GFPhi cells. (A) 500 GFPhi cells were injected into 16 recipient mice while 4000 or 10,000 GFPlo cells were injected into 8 mice per group. BLI activity was subsequently measured over six weeks and normalized to the signal at the time of injection. (B) Reconstitution ability was highly enriched in the GFPhi fraction, with successful reconstitution (green dots) in 9 out of 16 recipients, compared to 3/8 mice receiving 10,000 GFPlo cells and 1/8 mice receiving 4000 GFPlo cells. Overall, the 14 cases of reconstitution that were confirmed by alum carmine staining also represented the top14 final BLI readings. (9.30 MB TIF) [file pone.0008035.s001.tif]

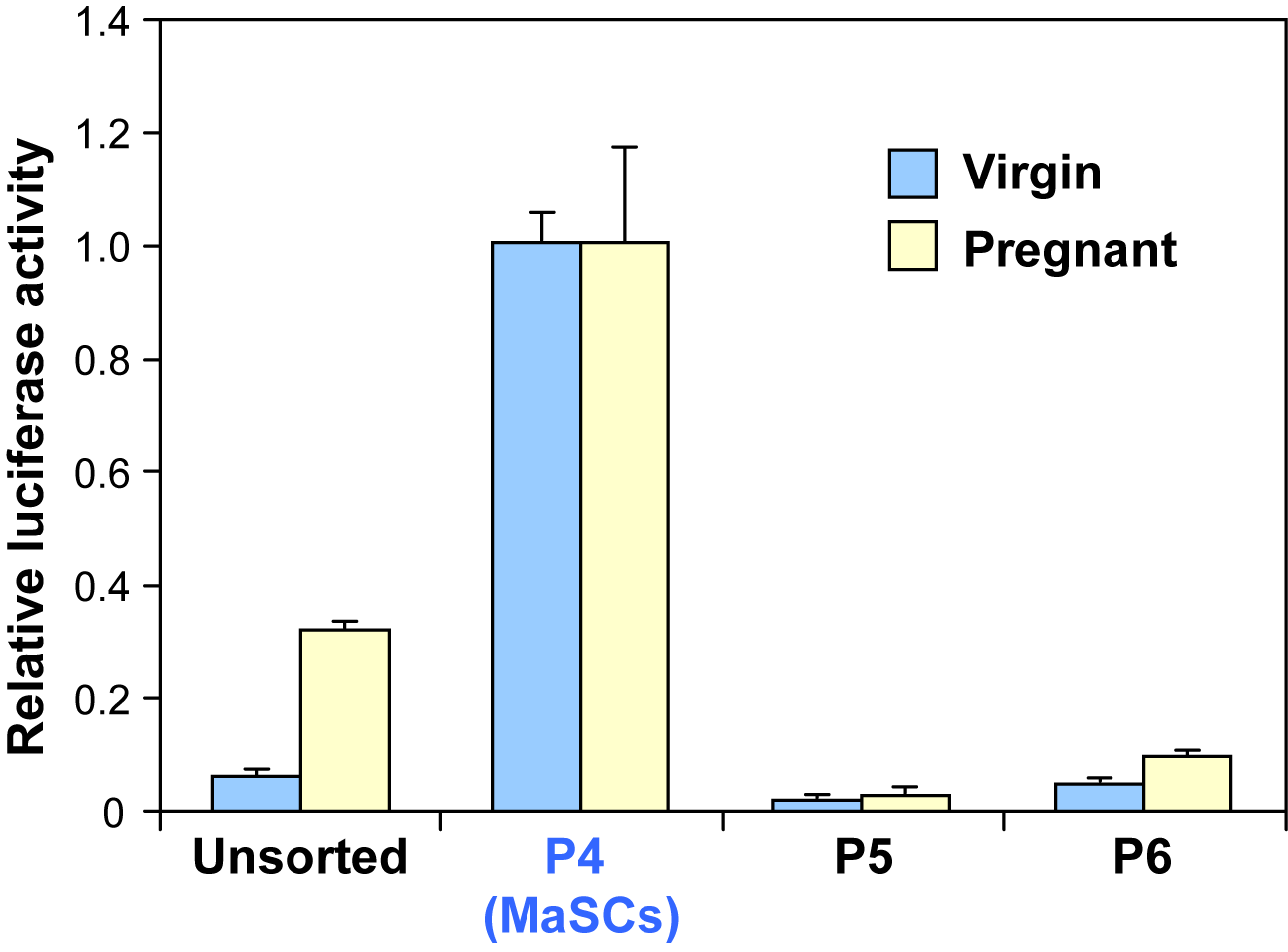

Supplement: Figure S2 — In vitro luciferase assay for sorted mammary epithelial cells from virgin and parous mice. Mammary epithelial cell populations were collected based on CD24 and CD29 staining and lysed for in vitro quantification of luciferase activity. Data shown is normalized to P4 cells (+/− SEM). The same pattern of luciferase activity is seen in virgin and pregnant mice, with the higher expression of luciferase in unsorted populations as the result of the higher percentage of luciferase expressing MaSCs in the parous mice. (3.76 MB TIF) [file pone.0008035.s002.tif]
